# Combination of enamel matrix derivative and hyaluronic acid inhibits lipopolysaccharide-induced inflammatory response on human epithelial and bone cells

**DOI:** 10.1007/s00784-021-04152-8

**Published:** 2021-08-30

**Authors:** Liza L. Ramenzoni, Laura Annasohn, Richard J. Miron, Thomas Attin, Patrick R. Schmidlin

**Affiliations:** 1 Clinic of Conservative and Preventive Dentistry, Center of Dental Medicine, University of Zurich, Zurich, Switzerland; 2 Laboratory of Applied Periodontal and Peri-Implantitis Sciences, Clinic of Conservative and Preventive Dentistry, Center of Dental Medicine, University of Zurich, Zurich, Switzerland; 3 Department of Periodontology, University of Bern, Bern, Switzerland

**Keywords:** Enamel matrix derivative, Hyaluronic acid, Cell viability, Bone regeneration, Oral wound healing, Pro-inflammatory cytokines

## Abstract

**Objectives:**

The aim of this study was to evaluate the in vitro effect of enamel matrix derivative (EMD) and hyaluronic acid (HA) and their synergistic combination on lipopolysaccharides (LPS)-induced inflammation in human keratinocytes and osteoblasts.

**Material and methods:**

Cells were challenged with LPS (1 μg/ml) and cultured in the following treatment groups with EMD (30 mg/ml) and HA (30 mg/ml): LPS, EMD, HA, EMD + HA, EMD + LPS, HA + LPS, and EMD + HA + LPS. Cell viability, inflammatory cytokine expression, and cell migration were determined using colorimetric assay, quantitative real-time polymerase chain reaction (qPCR), and scratch wound healing assay, respectively.

**Results:**

Cell viability was decreased when exposed to LPS compared to the controls. Overall, LPS treatment expressed upregulation on inflammatory cytokine tumor necrosis factor alpha (*TNF-α*), interleukin 1 beta (*IL-1β*), and interleukin 6 (*IL-6*). EMD and HA reduced up to 3.0-fold the cytokine expression caused by LPS (*p* < 0.05). EMD and HA statistically induced higher migration in osteoblasts and keratinocytes, respectively. Migration was impaired by LPS, whereas it significantly increased after addition of EMD and HA.

**Conclusions:**

EMD and HA are advantageous biomaterials that individually generate strong directional migratory keratinocyte and osteoblast response. Their combination also enhances cell viability, and anti-inflammatory and migratory abilities to promote healing specially under LPS inflammatory stimulus. Future in vivo and animal research is necessary to further characterize the effect of EMD and HA on periodontal regeneration.

**Clinical relevance:**

The use of EMD in conjunction with HA resulted in a reduction of inflammation and improvement of tissue healing at wound sites. Both biomaterials combined may potentially improve the effectiveness of bone regeneration in periodontal bone defects, pointing to the potential clinical relevance of both materials in regenerative periodontal surgery.

## Introduction

The major goals of periodontal therapy are generally recognized to be cessation of periodontal attachment loss and regeneration/reconstruction of lost periodontal tissues [1–4]. In this context, many surgical procedures, i.e., collagen barrier membranes and bone grafts, have been developed in order to achieve regeneration [5]. Despite the regenerative histological verification in surgical approaches, throughout full predictable clinical reconstruction is yet a challenging goal to be reached [3, 5]. In the past decades, many studies have concerned themselves with establishing biomaterials to favorably promote periodontal regeneration, in addition to surgery techniques [6, 7]. And a plethora of tissue growth/differentiation factors have already been shown to promote wound healing, anti-inflammation, and de novo tissue formation [8].

Enamel matrix derivative, amidst other many biological factors, has been widely verified as the go-to biomaterial for the purpose of obtaining both soft and hard tissue growth, such as periodontal ligament, cementum, and alveolar bone [9–14]. As a porcine fetal tooth extract, more than 90% of the total protein content of enamel matrix derivative (EMD) is composed of amelogenins and the remaining 10% includes enamelin, ameloblastin, amelotin, and apin [14]. Besides the beneficial effect of EMD on bone defects, it may indeed promote healing of soft tissue wounds by attenuating gingival inflammation [15, 16]. Nevertheless, the potential effects of EMD on inflammatory response and cytokine expression remain mostly unexplored. In addition, there are few reports focusing on defining the biologic mechanisms of the observed EMD anti-inflammatory effects [17]. In addition to EMD, hyaluronic acid (HA), known as non-sulfated glycosaminoglycan, has also been considered an ubiquitous optimal biomaterial for tissue regeneration due to its hygroscopic and viscoelastic properties [18, 19]. Given its broad expression in maintaining extracellular matrix of connective tissues, HA has been utilized in numerous tissue engineering biomedical applications, and has been confirmed to play a significant role in periodontium repair/regeneration and cell migration in mineralized/non-mineralized tissues [19, 20]. Non-crosslinked HA is considered biodegradable, biocompatible, and bioresorbable, and also well recognized to improve tissue lubrication in cartilage, to guide cell growth and differentiation, and to speed up the healing and repair of chronic wounds [21]. Additionally, HA has also been hypothesized to have an anti-inflammatory effect on soft and hard tissue healing, which may be of significance in periodontal regeneration [22, 23]. Interestingly, other in vitro studies showed that HA may prompt a bacteriostatic response by reducing periodontal pathogenic bacteria [23–26] and minimizing bacterial recolonization after mechanical debridement [27].

The application of EMD and HA as adjuvant chemotherapeutic bioagents in periodontal therapy is noteworthy; however, there are a limited number of studies investigating in vitro tissue wound healing response to typical pyrogen bacterial endotoxins, such as lipopolysaccharides (LPS), which induce detrimental biological responses and act as an important factor in periodontitis pathogenesis [28–31]. A number of other studies describe the use of HA and EMD in non-surgical and surgical periodontal therapy, with generally beneficial moderate effects on periodontal inflammation, on bleeding on probing, or residual pocket depth [11, 14, 20, 27–31]. Furthermore, different combinations of EMD and bone grafts and/or platelet growth factors have been used to regenerate intrabony defects [32]. Only few studies exist on the use of HA and EMD in regenerative periodontal surgery [18]. As such, it is still unclear to what extent the combination of EMD and HA may lead to additional tissue healing or inflammatory response compared to the use of EMD or HA alone. Before clinical studies are conducted to verify a EMD-HA compound benefit, a better understanding of its combined influence on the tissue-cell behavior involved in periodontal regeneration is still needed.

Therefore, the goal of the present study was to investigate the in vitro effects of EMD and HA preparations on LPS-induced sterile inflammation in human gingival keratinocytes and alveolar osteoblasts, as the main representative cell types involved in soft and hard tissue regeneration in the oral cavity. To investigate whether EMD and HA and their combination might modulate inflammatory response and wound healing, we have assessed the gene expression of cytokines and cell migration in human epithelial gingival keratinocytes (HEGK) and human alveolar osteoblast cells (HOAS) stimulated with LPS derived from the cell walls of gram‐negative *Porphyromonas gingivalis*. We hypothesized that the combination of EMD and HA works synergistically to positively stimulate the wound healing potential and reduce inflammation of oral gingival keratinocytes and osteoblasts in vitro and thus may collaboratively contribute to soft tissue healing/regeneration following reconstructive periodontal surgery.

## Materials and methods

### Cell culture

Primary human alveolar osteoblasts cells were donated by G.E.R.N. (Tissue Replacement, Regeneration & Neogenesis, Department of Operative Dentistry and Periodontology), Faculty of Medicine, University of Freiburg, Freiburg, Germany. Approval to conduct this cell study was granted by the Ethics Committee of the Albert-Ludwigs-University Freiburg for research involving humans (EK153-15) and informed written consent obtained from the donors, in accordance with the Declaration of Helsinki. Monolayers of osteoblasts were obtained by seeding the cells with Dulbecco’s minimal essential medium (DMEM, Invitrogen, Karlsbad, USA) supplemented with 10% fetal bovine serum (FBS, Invitrogen, Karlsbad, USA), 100 units penicillin, and 100 μg/ml streptomycin (Biochrom, Berlin, Germany) at 37 °C, 95% air, and 5% CO_2_. Osteoblast cells were derived from operative alveolar bone biopsies and obtained from 4 different patients undergoing corrective osteotomy surgery. These cells were expanded by splitting and used between passages 4th and 10th. Osteoblast cells were seeded (5 × 10^5^ cells/well) on cell culture flasks (T25 and T75, Merck KGaA, Darmstadt, Germany) and grown to 80% confluence. After confluence was achieved, the cells were washed with phosphate-buffered saline (PBS, Sigma Aldrich, St. Louis, MO, USA) and resuspended with 0.25% trypsin (Seromond Biochrom, Berlin, Germany) to enable further passage into 12- and/or 24-well plates. Immortalized HGEK-16 cells were previously described [33] and donated by the Oral Microbiology Institute, Clinic of Conservative and Preventive Dentistry, Center of Dental Medicine, University of Zurich, Zurich, Switzerland. HGEK cells were cultured in an incubator (5% CO_2_, 95% air at 37 °C) and passaged at regular intervals depending on their growth characteristics using 0.25% trypsin (Seromond Biochrom, Berlin, Germany) and maintained in complete epithelial medium consisting of defined keratinocyte serum free medium (Gibco, Life Technologies GmbH, Carlsruhe, Germany), supplemented with 100 U/ml penicillin and 100 mg/ml streptomycin (Sigma, St. Louis, Missouri, USA), 2 mM l-glutamine, and 0.25 mg/ml fungizone (Sigma, St. Louis, Missouri, USA). Change in cell culture medium and cell passage were conducted every 2 days using a new culture medium. The keratinocytes used in this study were between the 10th and 16th passages.

### Cell treatment with EMD, HA, and LPS

EMD was purchased from Institute Straumann (Institute Straumann AG, Basel, Switzerland). Following previous in vitro and in vivo studies [9–11, 34–37], the concentration of EMD at 30 mg/ml was chosen to be used for experimental seeding. EMD stock solutions were diluted in DMEM (Invitrogen, Karlsbad, USA) supplemented with 10% FBS (Invitrogen, Karlsbad, USA). HA was purchased from Regedent (Regedent AG, Zurich, Switzerland) utilizing one composition of non-cross linked native HA (HyaDENT, BioScience GmbH, Dümmer, Germany). In order to compare HA at the same concentrations to EMD and following previous in vitro studies [38–41], HA working solution was also chosen to be prepared in the same concentration of 30 mg/ml (same as EMD) prior to cell seeding experiments. In addition, purified *P. gingivalis* LPS (InvivoGen, San Diego, CA, USA) was directly diluted in culture medium to a final concentration of 1 μg/ml with the purpose of inducing inflammation [37, 42]. Control samples were seeded without reagents containing DMEM with 10% FBS and 1% antibiotics. HEGK and HOAS were cultured with their specific medium described above until reaching confluency and cultured for a further 7 days (medium replaced every 2 days). Then, each cell type was separately seeded (1 × 10^5^ cells/well) on 12-well or 24-well plates prior to treatment. Cells were exposed to 8 different conditions as follows: (1) control (untreated); (2) LPS (1 μg/ml); (3) EMD (30 mg/ml); (4) HA (30 mg/ml); (5) EMD (30 mg/ml) + HA (30 mg/ml); (6) EMD (30 mg/ml) + LPS (1 μg/ml); (7) HA (30 mg/ml) + LPS (1 μg/ml); (8) EMD (30 mg/ml) + HA (30 mg/ml) + LPS (1 μg/ml).

### Cell viability

The influence of EMD, HA, EMD + HA combination, and/or LPS on keratinocytes and osteoblast viability was determined by the nonradioactive, colorimetric MTT staining assay according to the manufacturer’s protocol (MTT: 3-[4,5-dimethylthiazol-2-yl]-2,5-diphenyltetrazolium bromide; Sigma-Aldrich, Steinheim, Germany) as previously described [43]. Tetrazolium bromide was fermented to formazan by viable cells and resulting formazan was measured after cell lysis photometrically. The keratinocytes and osteoblasts were separately seeded (1 × 10^5^ cells/well) on 24-well plates and treated for 24 h at 37 °C with the following: (1) control (untreated); (2) LPS (1 μg/ml); (3) EMD (30 mg/ml); (4) HA (30 mg/ml); (5) EMD (30 mg/ml) + HA (30 mg/ml); (6) EMD (30 mg/ml) + LPS (1 μg/ml); (7) HA (30 mg/ml) + LPS (1 μg/ml); (8) EMD (30 mg/ml) + HA (30 mg/ml) + LPS (1 μg/ml). After exposure, cells were washed with PBS (1 ×) once. Then, the solution of MTT (5 mg/ml in PBS 1 ×) was added to each well and the cells were incubated for further 4 h at 37 °C. After the incubation period, 1 ml of isopropanol solution (1 N HCl—isopropanol) was added to the cells as a solubilization reagent. Before measuring the samples in the spectrophotometer plate reader, mixture isopropanol was collected and centrifuged for 5 min at 600* g* force. Absorbance was taken by spectrophotometry reading at 570 nm with reference absorbance at 690 nm (Spectroquant Prove 300, Tecan, Austria, USA). In total, 3 independent experiments were conducted in triplicates.

### Gene expression analysis

To comprehensively assess the combined effect of EMD and HA on HEGK and HOAS induced with LPS towards a pro-inflammatory phenotype, real-time quantitative PCR (qPCR) gene expression analysis of classic inflammatory molecules was performed. Cells were exposed for 24 h to 8 previously described treatment groups above. Results were set in relation to differentiated cells cultured under exposure-free conditions as controls. Gene expression analysis at the mRNA level was performed for tumor necrosis factor-α (*TNFα*), interleukin-1β (*IL-1β*), and interleukin-6 (*IL-6*). Osteoblasts and keratinocytes were first seeded (1 × 10^5^ cells/well, 24-well plates) for at least 1 week to enable confluence. Then, the normal medium was replaced with treatment medium for 24 h containing different described concentrations: (1) control (untreated); (2) LPS (1 μg/ml); (3) EMD (30 mg/ml); (4) HA (30 mg/ml); (5) EMD (30 mg/ml) + HA (30 mg/ml); (6) EMD (30 mg/ml) + LPS (1 μg/ml); (7) HA (30 mg/ml) + LPS (1 μg/ml); (8) EMD (30 mg/ml) + HA (30 mg/ml) + LPS (1 μg/ml). Total mRNA (maximum 1 μg) was isolated with Trizol (Invitrogen, Grand Island, NY, USA) and converted into cDNA using the RevertAid First Strand cDNA Synthesis Kit (Roche, Basel, Switzerland). The quality and quantity of the isolated RNA were analyzed using a NanoDrop ND1000 spectrophotometer (Thermo Fisher Scientific, Waltham, Massachusetts, USA). The qPCR reactions were performed using the 7500 real-time PCR system (Applied Biosystems, Grand Island, New York, USA), the Power SYBR Green PCR Master Mix (Applied Biosystems, Grand Island, New York, USA), and cDNA equivalent to 30 ng total mRNA. Three independent experiments were performed for genes with the following specific primers (purchased from Microsynth AG, Balgach, Switzerland): *GAPDH* (forward primer: 5′-AAT CCC ATC ACC ATC TTC CA-3′, reverse primer: 5′-TGG ACT CCA CGA CGT ACT CA-3′), *TNFα* (forward primer: 5′-AGG CGC TCC CCA AGA AGA CA-3′, reverse primer: 5′-TCC TTG GCA AAA CTG CAC CT-3′), *IL-1β* (forward primer: 5′- ACA GAT GAA GTG CTC CTT CCA-3′, reverse primer: 5′- GTC GGA GAT TCG TAG CTG GAT -3′), and *IL-6* (forward primer: 5′-GGT ACA TCC TCG ACG GCA TCT-3′, reverse primer: 5′-GTG CCT CTT TGC TGC TTT CAC-3′). The relative mRNA expression of genes was normalized to the housekeeping gene *GADPH* and was analyzed using the comparative Ct method (2^−ΔΔCT^ formula). All samples were tested in triplicate and 3 independent experiments were performed. The results were presented in means ± standard deviations.

### Scratch wound healing migration assay

To determine the effect of EMD, HA, and LPS on wound healing, a scratch-wounded cell migration monolayer model was used [43]. The cells were seeded at a density of 1 × 10^5^ cells/ml and cultured into each well of a 24-well plate and incubated for 24 h at 37 °C until confluent. Prior to the scratch assay, the cells were exposed to 10 μg/ml of mitomycin C (Sigma-Aldrich, Steinheim, Germany) in serum-free media for 2 h, which inhibited mitosis of the cells. The wound was produced by scratching with a 10-μl pipette tip (700–900 μm in diameter). Following PBS 1 × washes to remove cell debris, the remaining adherent cells were divided in 8 treatment groups: (1) control (untreated); (2) LPS (1 μg/ml); (3) EMD (30 mg/ml); (4) HA (30 mg/ml); (5) EMD (30 mg/ml) + HA (30 mg/ml); (6) EMD (30 mg/ml) + LPS (1 μg/ml); (7) HA (30 mg/ml) + LPS (1 μg/ml); (8) EMD (30 mg/ml) + HA (30 mg/ml) + LPS (1 μg/ml). Digital images were captured during 24 h using a camera-equipped, inverted microscope (Carl Zeiss AG, Oberkochen, Germany) and wound width measurements were subtracted from wound width at time 0 to obtain the net wound closure. Initial wound edges marked the initial cell migration and were used to identify the decrease in wound width throughout the whole experiment. The distances between edges of injured monolayers were measured by the ImageJ software (National Institutes of Health, USA) in pixels and wound closure was expressed with comparison images captured between time points 0 and 24 h after wound simulation.

### Statistical analysis

The mean values and standard deviations were computed for the MTT test, and multiple comparison analysis of variance (ANOVA) with Bonferroni adjustment with a global significance level of 5% was conducted to assess the statistical significance of the differences between the experimental groups using IBM SPSS software (IBM SPSS Statistics for Windows, version 23.0; IBM Corp., Armonk, NY). Differences were considered significant at *p* < 0.05 and all experiments were performed in triplicate and repeated at least three times under the same conditions.

## Results

### Effect of EMD, HA, and LPS and their combinations on cell viability

**Fig. 1:**
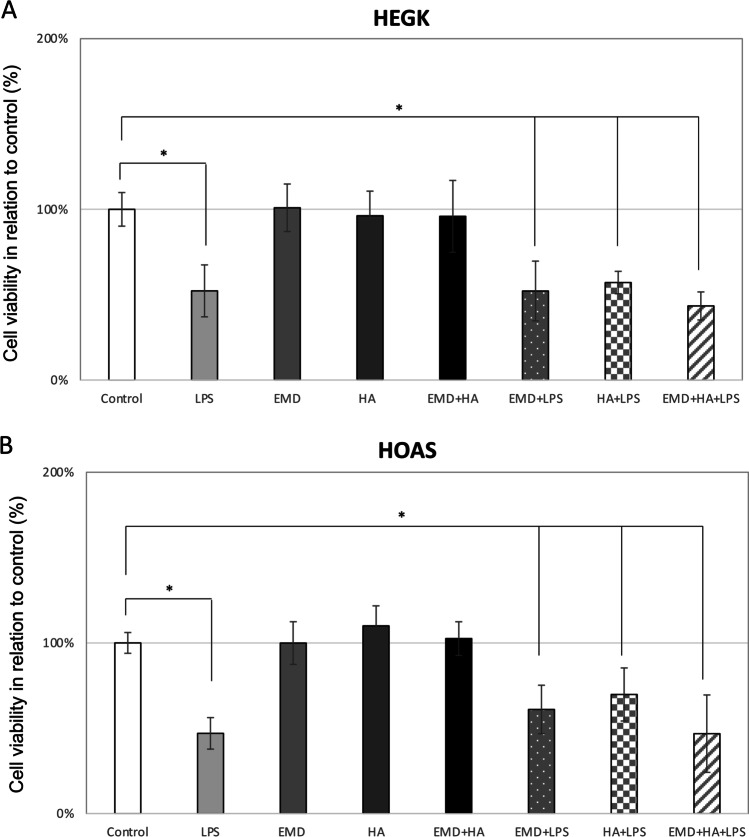
Evaluation of cellular viability of keratinocytes (HEGK) (**A**) and osteoblasts (HOAS) (**B**). Graphic bars represent the percentage, with respect to control cells (untreated, 100%), of viable cells after 24 h exposure treatment to: (1) LPS (1 μg/ml); (2) EMD (30 mg/ml); (3) HA (30 mg/ml); (4) EMD (30 mg/ml) + HA (30 mg/ml); (5) EMD (30 mg/ml) + LPS (1 μg/ml); (6) HA (30 mg/ml) + LPS (1 μg/ml); (7) EMD (30 mg/ml) + HA (30 mg/ml) + LPS (1 μg/ml). Data show the mean ± SE (*n* = 3). Statistically significant with respect to the control according to one-way ANOVA; * *p* < 0.05. *Y*-axis = optical density

To investigate cell viability, the MTT cell assay was used to determine the effects of EMD (30 mg/ml), HA (30 mg/ml), and LPS (1 μg/ml) on keratinocytes and osteoblasts cultured for 24 h in the following 8 treatment groups: (1) control (untreated); (2) LPS (1 μg/ml); (3) EMD (30 mg/ml); (4) HA (30 mg/ml); (5) EMD (30 mg/ml) + HA (30 mg/ml); (6) EMD (30 mg/ml) + LPS (1 μg/ml); (7) HA (30 mg/ml) + LPS (1 μg/ml); (8) EMD (30 mg/ml) + HA (30 mg/ml) + LPS (1 μg/ml). HEGK cell viability was severely decreased to up to 48% when exposed to LPS alone and preparations of EMD and/or HA mixed with LPS compared to the untreated negative control (*p* < 0.05, Fig. 1A). Treatment of EMD and HA and their combination showed no significant change in HEGK cell toxicity and they did not significantly affect cell numbers. Also, HA did not improve cell viability on HEGK exposed simultaneously to LPS (HA + LPS, *p* > 0.05, Fig. 1A). Similarly, LPS alone and preparations of EMD and/or HA mixed with LPS were found to be damaging for HOAS by reducing its viability (i.e., up to 53% for LPS alone). Cell numbers were also reduced compared to the untreated control (*p* < 0.05, Fig. 1B). A nonsignificant change in cell viability was observed after a 24-h incubation time with 30 mg/ml concentration of EMD and HA and a combination of EMD + HA without LPS treatment (*p* > 0.05, Fig. 1B). The present in vitro conditions indicated that EMD and HA were extremely biocompatible materials that supported both keratinocyte and osteoblast cell survival at 30 mg/ml concentration. However, they did not change the detrimental effect of LPS on the cell viability (*p* > 0.05, Fig. 1A and  B).

### Effect of EMD, HA, and LPS and their combinations on TNF-α, IL-1β, and IL-6 expression

**Fig. 2:**
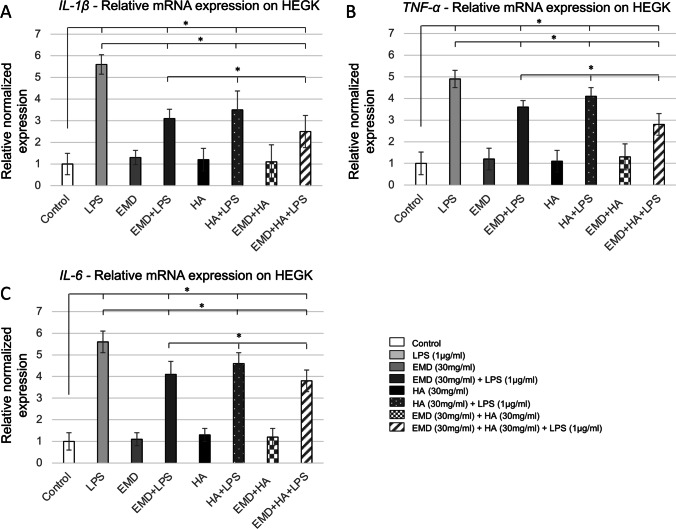
Expression of mRNA for inflammatory cytokines in HEGK challenged with EMD, HA, and LPS and their combinations. qPCR, with normalization to *GAPDH* using the Ct method analysis, are shown as means ± SD. **A** *IL-1β*; **B** *TNF-α*; **C** *IL-6*. The symbol * indicates a statistically significant increase in cytokine mRNA expression in comparison to non-challenged cells (two-way repeated measure ANOVA). * *p* < 0.05

**Fig. 3:**
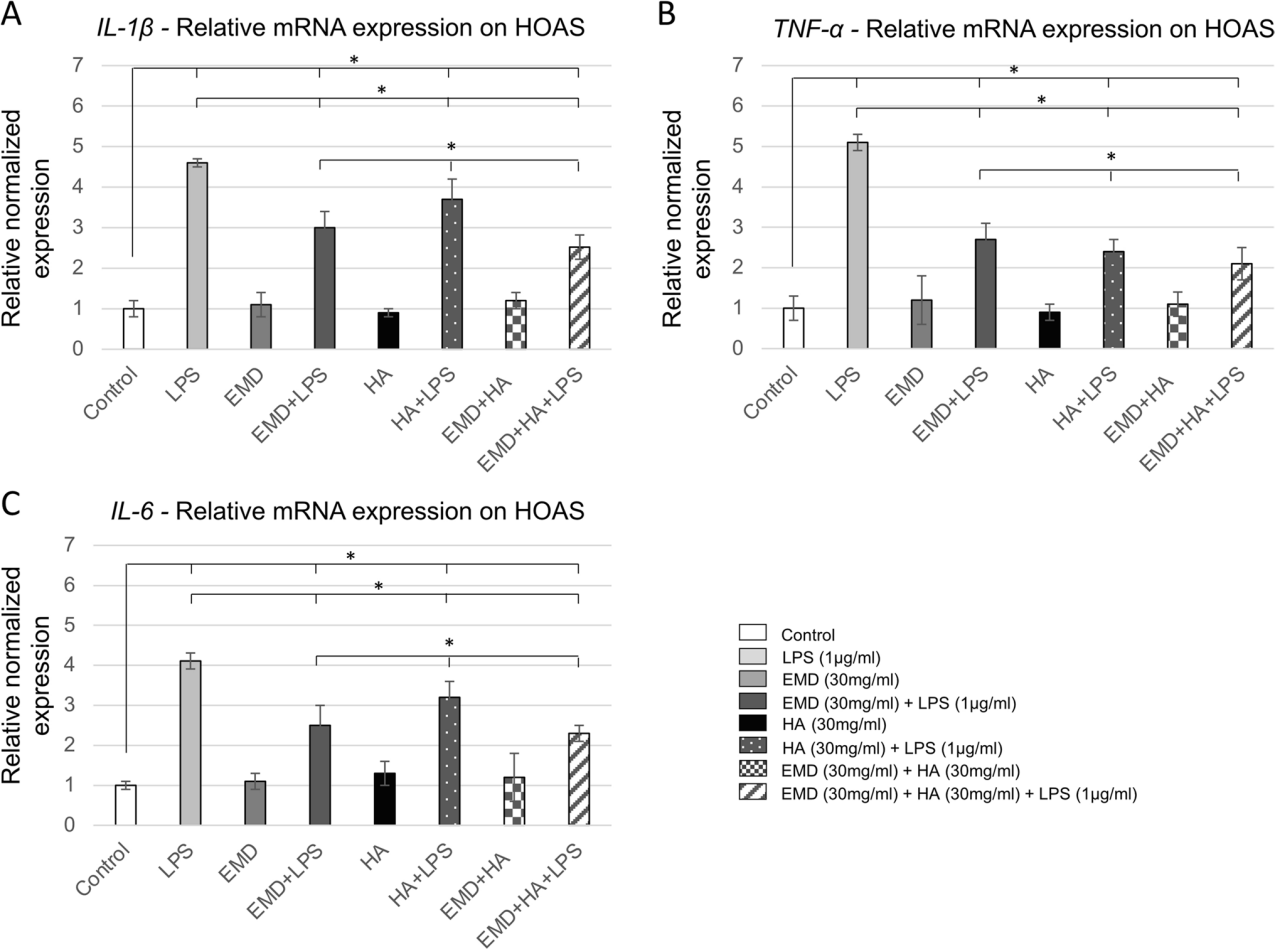
Expression of mRNA for inflammatory cytokines in HOAS challenged with EMD, HA, and LPS and their combinations. qPCR, with normalization to *GAPDH* using the Ct method analysis, are shown as means ± SD. (A) *IL-1β*; (B) *TNF-α*; (C) *IL-6*. The symbol ✽ indicates a statistically significant increase in cytokine mRNA expression in comparison to non-challenged cells (two-way repeated-measures ANOVA). * *p* < 0.05

Cultured HEGK challenged with LPS mixed with EMD and HA expressed increased mRNA levels for all inflammatory cytokines *TNF-α*, *IL-1β*, and *IL-6* (up to $\approx$ 5.5-fold upregulation) at 24 h compared to untreated controls (Fig. 2A, B, and C, *p* < 0.05, as indicated by the symbol *). The increase in pro-inflammatory gene expression was found to be comparatively similar to LPS alone for all cytokines. When treated with EMD and/or HA, the HEGK cells were able to reduce almost by half the pro-inflammatory gene expression caused by LPS (*p* < 0.05; two-way repeated-measures ANOVA) compared to LPS treatment alone (Fig. 2A, B, and C, *p* < 0.05, as indicated by the symbol *). In comparison to HEGK, HOAS cells demonstrated similar increase in mRNA levels for all inflammatory genes *TNF-α*, *IL-1β*, and *IL-6* ($\approx$ 3.5-fold upregulated) when treated with LPS at 24 h compared to untreated controls (Fig. 3A, B, and C, *p* < 0.05, as indicated by the symbol *). When treated with EMD and HA and their combination, the HOAS cells were also able to reduce pro-inflammatory gene expression caused by LPS by up to 2.5-fold (*p* < 0.05). The combination of EMD and HA further reduced the inflammatory cell response for both cell types when compared to separated EMD (EMD + LPS) and HA (HA + LPS) test group treatments (*p* < 0.05).

### Effect of EMD, HA, and LPS and their combinations on cell migration

**Fig. 4:**
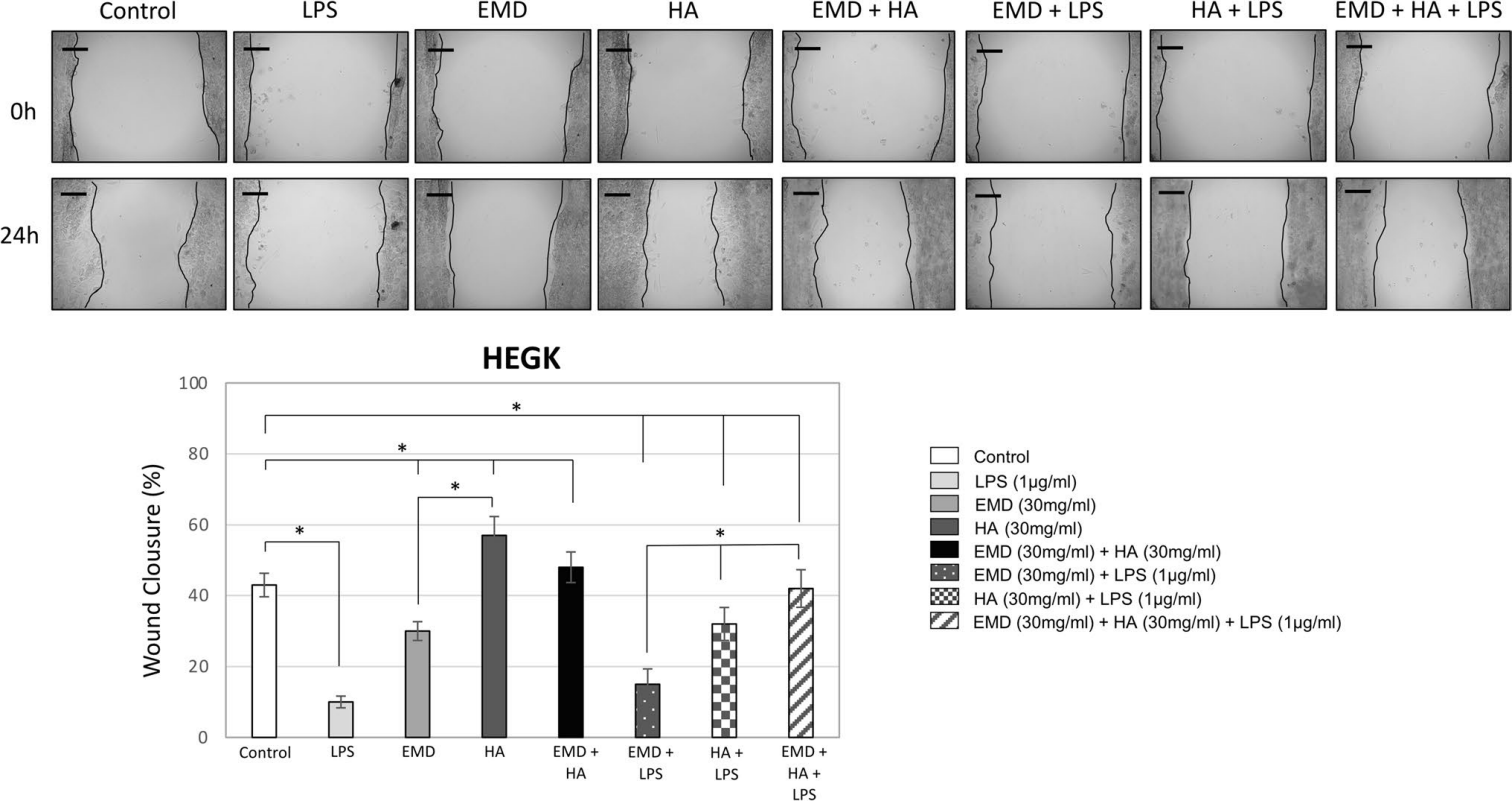
HEGK cell migration (scratch wound healing assay). Migratory ability of HEGK cells after culture with (1) LPS (1 μg/ml); (2) EMD (30 mg/ml); (3) HA (30 mg/ml); (4) EMD (30 mg/ml) + HA (30 mg/ml); (5) EMD (30 mg/ml) + LPS (1 μg/ml); (6) HA (30 mg/ml) + LPS (1 μg/ml); (7) EMD (30 mg/ml) + HA (30 mg/ml) + LPS (1 μg/ml). Images were recorded 24 h after wounding. Controls showed no or incomplete healing patterns and regarded as 0% wound closure. Representative images are shown from 3 independent experiments with brighter gray defined as areas lacking cells or wound area. ImageJ values of percentage wound closure mean ± SD, * *p* < 0.05. Scale bar: 50 μm

**Fig. 5:**
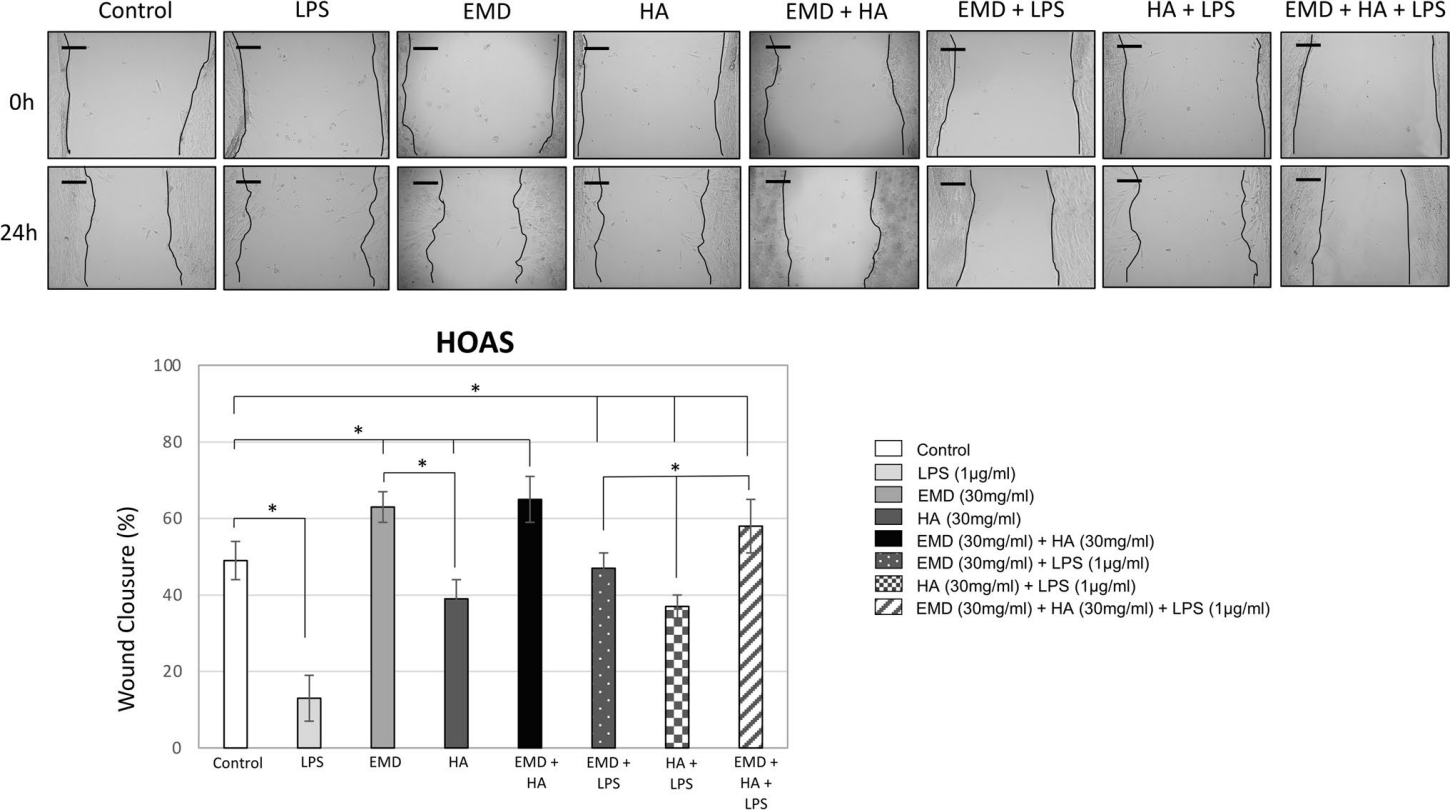
HOAS cell migration (scratch wound healing assay). Migratory ability of HEGK cells after 24 h culture with (1) LPS (1 μg/ml); (2) EMD (30 mg/ml); (3) HA (30 mg/ml); (4) EMD (30 mg/ml) + HA (30 mg/ml); (5) EMD (30 mg/ml) + LPS (1 μg/ml); (6) HA (30 mg/ml) + LPS (1 μg/ml); (7) EMD (30 mg/ml) + HA (30 mg/ml) + LPS (1 μg/ml). ImageJ values of percentage wound closure mean ± SD, * *p* < 0.05. Scale bar: 50 μm

After 24 h, both cell types HEGK presented increased migration compared to the untreated control and covered approximately 50 to 60% of the wound area when exposed to EMD and HA (Fig. 4). HEGK presented higher cell migration after treatment with HA when compared to EMD (Fig. 4, * *p* < 0.05). Significantly lower migration of HEGK (Fig. 4) and HOAS (Fig. 5) was observed in the groups overall exposed to LPS (LPS alone, HA + LPS, EMD + HA + LPS) compared to the untreated control group (* *p* < 0.05). At 24 h, a significant increase in the migration distance was observed for HEGK when exposed to HA + LPS compared to the exposure of LPS alone, which was not shown as significantly high as HEGK treated with EMD + LPS (Fig. 4, * *p* < 0.05). Conversely, HOAS increased their migratory ability after exposure to EMD + LPS compared to the exposure of LPS alone and the same effect was not found when treated with HA (Fig. 5, * *p* < 0.05). HOAS exhibited higher migration after treatment with EMD when compared to HA (Fig. 5, * *p* < 0.05). For both HEGK and HOAS, there was higher migration with groups treated with combined EMD + HA materials when simultaneously exposed to LPS and compared to separated EMD (EMD + LPS) and HA (HA + LPS) test group treatments (Figs. 4 and 5, * *p* < 0.05).

## Discussion

The purpose of this study was to determine the discriminant validity of using a combination of the regenerative biomaterials EMD and HA to reduce the pro-inflammatory effect of bacterial endotoxins, in addition to advancing wound healing in gingival and alveolar bone cells. This study is the first to report the beneficial combination of EMD and HA on the reduction of cytokine production and improvement of migration in response to induced-LPS inflammation. Specifically, we ascertained the effect of EMD and HA and their combination on the modulation of cytokine release induced by LPS from *P. gingivalis*, by attenuating the expression of pro-inflammatory cytokine (*TNF-α*) and chemokines (*IL-1β* and *IL-6*). In addition, LPS‐stimulated keratinocytes and osteoblasts treated simultaneously with EMD and HA decreased pro-inflammation and stimulated cell migration beyond that seen with EMD and HA alone. This inflammation decrease was observed in previous studies, where EMD and HA had been used separately as regenerative biomaterials in tandem with standard periodontal non-surgical and surgical treatments [6, 7, 14, 16]. A plethora of authors also have reported that EMD and HA are both capable of assisting the regeneration of periodontal tissues or the growth of the periodontal ligament, root cementum, and alveolar bone [5, 8, 11, 14, 16, 21, 27, 44]. As confirmed here, the osteoblasts and keratinocytes in fact presented a significant increase in cell migration when exposed to EMD and HA, respectively. It is also confirmed that EMD and HA may markedly improve clinical attachment levels, and reduce probing depth and inflammatory parameters [34, 41]. In several clinical trials, patients reported significantly less post‐treatment discomfort compared to conventional treatment [18, 44–47]. EMD and HA have also demonstrated anti-inflammatory properties clinically and in vitro [17, 23, 28, 29], which corroborates with our hypothesis. However, other reported potential mechanisms were less supported by our data. For example, the biologic mechanisms contributing to the effect of EMD and HA on inflammation remain controversial, since EMD was once shown to report external inflammatory root resorption or no significant differences in inflammatory mediators resulting from EMD application in the non‐surgical treatment of chronic periodontitis [48, 49].

Pro-inflammatory cytokines and chemokines, comprising *IL-1 β*, *IL-6*, and *TNF-α*, create an environment that fosters periodontal disease progression by affecting the balance in chronic inflammation [50]. The bacterial wall components, such as LPS, have the ability to induce a significant outbreak of pro-inflammatory cytokines from peripheral blood mononuclear cells and tissue macrophages in the periodontal tissue. The pro-inflammatory cytokine, *TNF‐α*, is elevated in early stages of periodontal inflammation and it is the main mediator of a number of inflammatory responses, stimulating cell apoptosis, increasing vascular permeability, inducing other cytokines and chemokines, and contributing to the recruitment of polymorphonuclear leukocytes [50, 51]. *TNF‐α* further stimulates osteoclast differentiation and activation, enhancing matrix metalloproteinases synthesis involved in soft tissue degradation [51]. Both *IL-6* and *IL-β* activate neutrophils, which cause chemotaxis and exocytosis, and consequently induce periodontal inflammation and tissue destruction [52]. In this study, we showed that the combination of EMD and HA has favorably reduced LPS‐mediated keratinocyte and osteoblast *IL-1 β*, *IL-6*, and *TNF‐α* production, indicating a potential mechanism for mediating their negative influence on periodontal wound‐healing process. This combination of EMD and HA benefited more than the use of EMD or HA alone under LPS treatment. Thus, the ability of EMD and HA to regulate inflammatory cytokine mediators could explain the improvement of the cell wound‐healing migration process in this study and implies a role for EMD and HA as immunomodulatory agents. In addition, bacterial infection is known to provoke oral tissue damage with rapid response of gingival fibroblasts against bacterial LPS [53]. And in vitro cell mobility is increased due to early gingival cell response to LPS by incremented HA synthesis [53, 54]. After LPS stimulation, the increase in HA was shown to be mediated by induction of prostaglandin-E2 synthesis in fibroblasts. High concentrations of LPS (> 1 μg/ml) effectively inhibit the proliferation of gingival cells and presumably reduce periodontal wound healing [37, 42, 53, 54]. The LPS repression may also be detrimental to keratinocyte proliferation, but as shown in the present study, this inhibition was partially overcome by the addition of combined EMD and HA. Pro-inflammatory activation caused by LPS reduces gingival cell motility in the early stages of inflammation and elevates *IL-6* and *TNF‐α* production in early stages of periodontal inflammation. EMD also appears to exert an influence on soft tissue cells that is compatible with improved wound healing and significantly downregulates the expression of *IL‐1β* and *cyclooxygenase‐2* [55, 56], which is confirmed by reduction of other cytokines in oral gingival keratinocytes here.

This study analyzed the mechanistic immunological impact of EMD combined with HA on limiting the release of pro-inflammatory cytokines induced by bacterial LPS. The anti-inflammatory potential of EMD and HA has been only minimally studied and the effect of their combination has been practically non-existent, which shows the importance and implication of in vitro studies for future use of biomaterial combinations in the clinical practice. The limitations of this in vitro study with relevance to clinical application of EMD combined to HA therapy are recognized and our in vitro experiments have certain limitations that can only be addressed by in vivo studies. The keratinocyte and osteoblast cells used in this study are the largest proportion of cells found in the periodontium but are not the only components. The presence of other tissues in the periodontal ligament will influence the effect of EMD-HA on overall homeostasis in vivo. Despite these clear limitations, the obtained in vitro results demonstrate that EMD in combination with HA could represent an additional tool to add to non-surgical periodontal therapy thanks also to their ability to reduce plaque and the growth of periodontopathogenic bacteria. In addition, the cellular mechanisms by which EMD and HA may modulate inflammation are mostly unknown and they underline the need for a more nuanced and sophisticated framework of further empirical studies. Further investigation should be taken, for example, to elucidate the roles of specific low molecular weight EMD amelogenins in modulating innate immune responses in periodontitis as well as the clinical potential of the hyaluronic acid preparations for oral soft tissue regeneration.

Within their limits, the present findings indicate that EMD and HA demonstrates synergistic inhibitory effect on LPS-induced inflammatory response by reducing proinflammatory cytokines and consequently improving wound healing migration. Interestingly, with the combination of EMD and HA, the expression of early pro-inflammatory cytokines was shown to be significantly reduced. Future animal and in vitro three-dimensional studies are still necessary to further investigate and provide evidence of the regeneration potential at the cell interaction level, before clinical application can be considered.
